# Spatial navigation in autism spectrum disorders: a critical review

**DOI:** 10.3389/fpsyg.2015.00031

**Published:** 2015-01-23

**Authors:** Alastair D. Smith

**Affiliations:** School of Psychology, University of Nottingham, Nottingham, UK

**Keywords:** autism, navigation, spatial, learning, cognition

## Abstract

On the basis of relative strengths that have been attributed to the autistic cognitive profile, it has been suggested by a number of theorists that people with autism spectrum disorders (ASD) excel at spatial navigational tasks. However, many of these claims have been made in the absence of a close inspection of extant data in the scientific literature, let alone anecdotal reports of daily navigational experiences. The present review gathers together published studies that have attempted to explicitly address functional components of navigation in ASD populations, including assays of wayfinding, large-scale search, and path integration. This inspection reveals a pattern of apparent strengths and weaknesses in navigational abilities, thus illustrating the necessity for a more measured and comprehensive approach to the understanding of spatial behavior in ASD.

## INTRODUCTION

Autism spectrum disorder (ASD) is a neurodevelopmental condition that is identified by difficulties with social communication and a restricted range of behavioral patterns and interests (American Psychiatric Association, 2013). Although the condition is characterized by these behavioral manifestations, ASD is increasingly described in the scientific literature with specific reference to perceptual and cognitive mechanisms (for a review, see Rajendran and Mitchell, 2007). Those that have received the most theoretical attention are a preference for processing local detail (Weak Central Coherence; Frith and Happé, 1994), difficulty understanding the perspective of others (Theory of Mind; Baron-Cohen, 1989), and impairments of executive tasks such as planning and inhibition (Executive Dysfunction; Ozonoff et al., 1991).

Compared to the study of other developmental conditions, it is a notable idiosyncrasy of autism research that some of the behavioral factors associated with ASD have been described as a relative strength of the cognitive profile. So, for example, whilst behaviors linked to weak central coherence may not necessarily favor an appreciation of the Gestalt, they might also result in superior detection of local details, compared to typically developing (TD) participants (Shah and Frith, 1983, 1993). Indeed, it is generally within the visuospatial domain that people with ASD have been shown to excel, and previous cohort studies have reported group effects that include increased efficiency in visual search, superior perception of slant, and a resistance to visual illusions (for a particularly thorough review and discussion of these findings and others see; Simmons et al., 2009). Such patterns have led Mottron and Burack (2001) and Mottron et al. (2006) to develop the theory of Enhanced Perceptual Functioning, which offers a neural explanation for peaks in performance across low-level perceptual tasks.

The framing of behaviors as relative strengths has since affected the way in which theorists discuss many aspects of autistic performance. This has been particularly apparent when it comes to discussions of spatial navigation, and there have been a number of claims that the strengths observed in small-scale laboratory tasks extend to large-scale everyday wayfinding. Some arguments have been made relatively incidentally, whereas others have been posited in specific treatises. An example of the former comes from Baron-Cohen (2008) who, when discussing his Hypersystemising theory of ASD, stated that strong systemisers “*would have had greater success in both using and making tools for hunting, or navigating space to explore far afield*” (p. 67). This argument is couched in evolutionary terms: proficiency in spatial tasks, and in understanding rule-based systems, is adaptive and thus would have conferred benefits upon our ancestors. However, Baron-Cohen (2008) does not expand upon this point, and we may therefore take it as an example of the potential breadth of the Hypersystemising account. In comparison, however, Reser (2011) very explicitly argues that the genes associated with ASD have been selected for, precisely because the condition confers a specific advantage for navigating large-scale environments. The “Solitary Forager Hypothesis” states that the autistic behavioral profile is ideal for foraging in the ancestral environment—preoccupations would be focused on the procurement of food, asocial tendencies would foster a desire to independently explore further afield, and an engagement in repetitive activities would be well suited to scanning the environment and picking up food.

Neither of the above accounts presents any empirical evidence for their claims that autistic individuals are good navigators, and yet it is not difficult to appreciate why one might reach such a conclusion: ASD is indeed associated with peaks in some visuospatial tasks, and systemising behaviors could conceivably lend themselves to an efficient analysis of regularities or changes in the surrounding environment. However, there are two core problems with this position. First, many anecdotal reports from people with ASD and their carers actually attest to a difficulty with daily navigation, and there are myriad accounts on internet forums of people with ASD being unable to find where they parked their car, or becoming lost in their home town because a familiar route was blocked. Not only do these difficulties affect the quality of everyday life, but they are also often the cause of anxiety, frustration, and embarrassment. The second key issue is that the extant scientific literature on navigation in ASD does not present a uniform picture of navigational prowess. Instead, there appear to be some components of navigational behavior in which people with ASD perform similarly to matched controls, some in which they appear to show a relative strength, and others in which they demonstrate a notable weakness. It is this literature that I explore in the present review, in the hope of producing a more accurate overview of current knowledge, and one that is firmly based upon empirical enquiry.

Spatial navigation is a complex behavior that is subject to great individual differences across typical and atypically developing people (Wolbers and Hegarty, 2010). Such differences result from the fact that effective navigation relies on a wide variety of processes, from moment-to-moment idiothetic (i.e., self-movement information) coding of positional change to enduring long-term representations of places and their configural relationships (e.g., Siegel and White, 1975; Golledge et al., 1985; Aguirre and D’Esposito, 1999; Hegarty et al., 2006). As a result, it is not surprising that there has been no comprehensive investigation of autistic navigation across all of these factors. However, although small, the current literature on navigation in ASD does cover a variety of skills, including path integration, large-scale search, and map utilization. In this review I have restricted inclusion only to published studies that have explicitly tested one or more of these factors in adults and children with ASD. This, therefore, does not include rodent models of autistic navigation (e.g., Crawley, 2007; Moy et al., 2007), nor does it include studies of autistic behavior on small-scale tasks that could arguably be related to large-scale navigational performance (e.g., Caron et al., 2006; Steele et al., 2007). Of the studies reviewed here, some were explicitly designed to test components of navigation, whereas others included navigational tasks within a more general exploration of spatial behavior. As a result, there are bound to be other examples in the literature that have evaded search owing to the fact that they were not intended to be studies of navigation *per se*.

## PATH INTEGRATION

Path integration is the process by which humans and non-human animals update their sense of position by use of idiothetic information, derived from labyrinthine and musculoskeletal sources. This process has been widely studied in adults (for a review, see Loomis et al., 1999), although much less so in development (Rieser and Pick, 2007; Smith et al., 2013). One component of path integration behavior is the ability to walk without vision to a previously-seen target location, and a study by Giovannini et al. (2009) explored this in a sample of children with ASD. The main theoretical drive of their study was to assess the dissociability of vision-for-locomotion and vision-for-perception in development, and Experiment 2 of their study included an autistic sample on the basis that a localist bias may give rise to an atypical profile (although there were no clear predictions about how this might be manifest in behavior).

Fifteen high-functioning children with ASD (mean age: 10 years) were compared to 18 TD children (mean age: 7 years), who were matched on performance IQ (PIQ) by use of Raven’s Colored Progressive Matrices (RCPM; Raven et al., 1962). An additional group of 10 adults without ASD was recruited for comparison with both developmental samples, and all participants completed two tasks. In the first, designed to assess vision-for-locomotion, participants were blindfolded and placed at a fixed starting position in a large indoor space. A target (orange cardboard disk) was then placed somewhere between 3 and 6 m from the participants, and the blindfold was removed for 3 s. The blindfold was then replaced and participants were asked to walk to where they remembered the target (which had since been removed) being positioned. Participants produced only one response. Their landing positions were converted into proportional signed errors and compared between groups—here there was found to be no statistical difference between any of the groups, with error being close to 0 for each of them. However, inspection of the figure provided by the authors does suggest that, on the whole, participants with ASD were a little more likely to undershoot the target location than the other groups.

In the second task, designed to assess vision-for-perception, participants were seated and asked to look at the target disk, which was again placed 3–6 m away from them. A rope was laid on the floor, reaching from the chair to the target, and participants were asked to adjust a similar rope (i.e., a tape measure that was gradually lengthened by an experimenter) until its length matched that of the first rope. This second rope was oriented orthogonally to the first, so that participants were adjusting the horizontal arm of an L-shape so that it matched the vertical arm. Again, participants only performed one trial of this task, and responses were also converted into a proportional signed error. In this task, children with ASD were significantly more accurate than both adults and TD children, who both tended to undershoot the target length (whereas ASD responses were close to 0). Errors across the two tasks did not correlate across participants, and RCPM did not predict children’s performance on either measure once age had been controlled for.

These data suggest that there is no difference between children with ASD and TD adults or children on a locomotion task that partly relies upon path integration processes. This may therefore mean that people with ASD are able to use vision-for-locomotion in the same way as TD people, and may also use idiothetic information to update location just as readily as TD participants. With regards to vision-for-perception, the Giovannini et al. (2009) data provide an interesting large-scale addition to the literature that demonstrates greater accuracy in ASD participants on some perceptual tasks. This may be a result of a localist bias in participants, which has been argued to render them “immune” from illusory scaling errors in horizontal/vertical matching tasks (see Happé, 1996; Ropar and Mitchell, 1999). Together, these findings argue for a dissociation between visual processes (i.e., perception vs. action), although our conclusions on ASD performance must be limited by the fact that participants only produced one response on either measure, and the parameters of the tasks were not systematically manipulated. Furthermore, one cannot extend these findings to path integration proper until a more rigorous assay has been employed, such as the triangle-completion task (see Smith et al., 2013).

## LARGE-SCALE SEARCH

Laboratory studies of visual search behavior have previously demonstrated greater efficiency in ASD groups on certain components of the task (e.g., O’Riordan et al., 2001; Jarrold et al., 2005). Pellicano et al. (2011) assessed whether this strength extends to large-scale navigational search behavior—i.e., a foraging task that utilized full body movements in 3D space, as opposed to eye movements directed to 2D stimuli on a monitor. They devised a task in which participants were required to search for a hidden target item by inspecting a number of potential locations. In this case, the location of the hidden target was probabilistically determined such that it was more likely to appear on one half of the display. Since the Hypersystemising account of ASD (see Baron-Cohen, 2008) highlights that people with the condition are more likely to systematically test different hypotheses, and will therefore more readily derive systems and patterns in the world around them, it was predicted that people with ASD would be more likely to learn the probabilistic cue and search more efficiently over time.

Twenty children with ASD (mean age: 10 years) were compared to a group of twenty TD children who were matched on chronological age, verbal IQ (VIQ, as measured by the British Picture Vocabulary Scale—BPVS; Dunn and Dunn, 2009) and PIQ (measured using RCPM). The experiment was conducted in a 4 m × 4 m chamber containing a raised platform floor. Embedded into the floor was a concentric array of 49 circular switches, each surrounded by an annulus of light-emitting diodes. The laboratory was dimly lit and surrounded by dark featureless curtains, thus removing any obvious landmarks other than the search locations. In a trial, 16 search locations were illuminated (green), with eight locations either side of the midline. This array was randomized for each participant and remained fixed throughout the experiment. Participants began their search at a fixed starting point at the edge on the array, on the midline, and were required to press the switch at each location until they had found the target (the light that turned from green to red when the switch was activated). There were 40 search trials, organized in two blocks of 20, and the target appeared on one side of the midline for 80% of the trials (equally across blocks). Children were instructed that the target was always present and that they were to find it as quickly as possible, but there was no mention of the uneven distribution.

In order to ascertain whether children with ASD demonstrated greater sensitivity to the probability cue, Pellicano et al. (2011) compared the density of inspections that were directed to the cued and uncued hemifields. They found that TD children were more likely than ASD children to search in the cued hemifield in the first block, although there was no difference between groups for block 2. This suggested that children with ASD were, in fact, taking longer to infer the rule than TD children. The authors also modeled the quality of the search patterns taken by children, deriving indices of both optimality (i.e., taking the shortest path to the target whilst inspecting all intervening locations) and systematicity (i.e., the consistency of search paths, irrespective of target location). On both of these measures, children with ASD were found to perform statistically lower than TD children—that is, their search paths were less optimal and less systematic. It was also found that children with ASD made reliably more revisits to previously-inspected locations than TD children, which may have been an index of poorer spatial memory, or a product of sub-optimal search paths. Interestingly, it was found that this revisit behavior was predicted by performance on other tasks: children with poorer visuo-spatial working memory (as measured by Corsi blocks) made more revisits, as did children with greater efficiency on an attention-to-detail task (the Children’s Embedded Figures Test; Witkin, 1971).

The results of the Pellicano et al. (2011) study suggest that the strengths that have been observed in small-scale search tasks do not necessarily extend to large-scale search behavior. Indeed, contrary to expectations, children with ASD were found to be less efficient at the task than TD participants. Furthermore, the predictions of the Hypersystemising theory were not met, as children with ASD explored the space in a manner that was less systematic than their TD counterparts. The fact that search efficiency was related to visuospatial working memory does point to a factor that could underlie these differences. Furthermore, a similar relationship with embedded figures performance suggests that configural processing may also play a part in tasks such as this (i.e., children may be less likely to assign probabilistic weight to a half of the array if the array itself has not been strongly configured into a whole). It is also interesting to note that a recent study of probability cueing in visual search found that children with ASD were as sensitive to cues as TD children (Jiang et al., 2013), which suggests that spatial learning does not necessarily operate in the same manner across scales (see Smith et al., 2010).

## VIRTUAL WAYFINDING

Perhaps the most widely used paradigm in modern navigational research is virtual wayfinding, where participants are required to learn a novel 3D environment that is presented via computer software. The great advantage of this technique is that it affords scientists a great deal of control over the environments that they present. Thus, one can present very simple spaces (such as a virtual Morris Maze), or much more detailed naturalistic contexts, without the difficulties that can be associated with real-world testing. In line with this range of potentials, some scientists have used virtual wayfinding tasks to study discrete components of place learning in ASD, whereas others have used them to study more complex components of everyday urban navigation.

An example of the former study comes from Edgin and Pennington (2005), who explored the profile of spatial cognition with reference to central coherence and executive function theories of ASD. Twenty-four high functioning children with ASD (mean age: 11 years) were compared to a group of TD children matched on chronological age, VIQ (measured by the Peabody Picture Vocabulary Test; Dunn et al., 1965) and PIQ (measured by the Block Design task from the Wechsler Intelligence Scale for Children; Wechsler, 1974). They presented participants with a battery of tasks that mostly consisted of small-scale measures, such as the embedded figures test, and a spatial working memory test. However, it also included a virtual navigation task in the form of a computerized Morris Water Maze. In this experiment, based on a classic test of rodent navigation, children were required to learn and remember a hidden spatial location. Using a joystick, children navigated themselves around a circular arena, which was oriented by extra-maze cues in the form of a square chamber beyond the circular boundary, with distinguishing markers on each of the walls. A blue rug was visible on the floor and children were first required to navigate to that rug for a number of acquisition trials. They were then told that the rug had become invisible, and that they must search the arena to find it. There were five probe trials, each beginning from a different point around the circumference of the maze, and once the child had moved to the location containing the rug it became visible to them. The authors measured the amount of time that children spent searching in the correct quadrant of the maze, a measure of successful place learning, and found no group difference between ASD and TD children. However, they did find that there was no relationship between ASD performance and chronological age, whereas older TD children demonstrated improved spatial learning compared to younger TD children. This suggests that the rate of development for this behavior is slower in ASD; although, as noted by the authors, such a conclusion must be limited by the relatively restricted age range tested. Edgin and Pennington (2005) did find a difference in embedded figures performance, in line with previous demonstrations of more efficient behavior in participants with ASD, but not in any of their other spatial measures, suggesting that children with ASD were not generally superior across the domain of spatial behaviors tested.

Lind et al. (2013) used a more complex virtual environment to measure the navigational skills of a sample of adults with ASD. Some theorists have highlighted the role of episodic memory in efficient navigation (e.g., Hassabis et al., 2007; Spiers and Maguire, 2008), whereas others have favored an explanation that places the ability to appreciate alternative perspectives at the heart of navigation (Buckner and Carroll, 2007). Since these are both processes that have been shown to be impaired in people with ASD, Lind et al. (2013) predicted that performance in a navigational task that is based upon survey knowledge (i.e., an allocentric viewer-independent representation) would be poorer in participants with autism. This, in turn, would be counter to the predictions of the Hypersystemising theory of ASD. Twenty-seven high-functioning adults with ASD (mean age: 35 years) were tested alongside a group of neurotypical adults matched on chronological age, VIQ, PIQ, and full-scale IQ (all measured using the Wechsler Abbreviated Scale of Intelligence; Wechsler, 1999). Participants completed the Memory Island task, whereupon they used a joystick to explore a richly-textured virtual island in search of a “mysterious object.” The island contained a variety of naturalistic landmarks (as well as audio content) and participants began each trial in its centre (although facing different directs). In the first phase of the experiment, the locations of four objects (one per trial) were clearly marked by flags, and participants were asked to navigate to the flag in order to locate the object. Once they had located it, they were instructed to remember its location. This phase relied upon basic locomotor guidance in order to find the object, but also required participants to construct a cognitive map for the environment in order to remember the object locations. The quality of this representation was assessed in the second phase, where participants were instructed to navigate to each of the objects (one per trial) without the presence of the flag markers.

The authors took a number of dependent measures, including the proportion of time spent in the correct quadrant of the island, trials latency, movement velocity, path length, and the proportion of trials in which the object was located within 2 min (after which assistance was provided). They found that participants with ASD spent significantly less time in the correct quadrant when the target was hidden, compared to when it was signified by a flag. In comparison, typical adults spent an equal amount of time in the correct area in both phases. Lind et al. (2013) also found significant group differences in other measures, with ASD participants demonstrating longer latencies, slower velocity, and fewer successful trials. Critically, these differences occurred in both phases of the task, indicating a general diminishment of navigational efficiency in the ASD group, irrespective of whether they were locomoting toward a beacon or navigating on the basis of a cognitive map. The performance of both groups was related to scores on other measures of episodic memory and Theory of Mind, although there were no meaningful differences between groups in terms of these associations. Overall, these findings therefore contrasted with those of Edgin and Pennington (2005) by showing that participants with ASD performed reliably poorer than control participants—they were generally less efficient, but were also exploring incorrect parts of the island when searching for target locations.

A somewhat similar task was employed by Fornasari et al. (2013) in a study of urban wayfinding. They compared a group of 16 high-functioning children with ASD (mean age: 10 years) to a TD control group matched on chronological age, race, sex, language and educational level. Groups were not matched on measures of VIQ or PIQ, and the authors went on to examine participants on a battery of performance measures (e.g., RCPM, block construction, figure copying). This included the Route Finding component of the of the NEPSY-II battery (Korkman et al., 2007), where children are shown a schematic map containing a house and are then required to locate the house within a larger map (containing other houses and streets). Children with ASD performed poorer than their TD counterparts on all of these measures, demonstrating lower PIQ and relatively impaired visuospatial abilities. The authors then compared groups on a virtual wayfinding task, where children used a mouse to control exploration around an unfamiliar urban environment. After a familiarization stage (in a simple courtyard space) children were presented with a small town environment, consisting of streets and buildings that formed 11 different zones. There were two phases to the task, and in the first children were asked to freely explore the town until they thought that they had seen it in its entirety. In the second phase, the children were shown an object and told that there were five of them to search for throughout the town. Children then performed this “treasure hunt” and clicked on each of the objects when they had been located—an onscreen running total reminded them of how many remained.

Fornasari et al. (2013) took a number of dependent measures of exploration in both phases, including the number of different zones visited, the number of revisits to locations already explored, and properties of the exploration movements (e.g., path length, time spent stationary, or moving). These data were analyzed with RCPM as a covariate, in order to control for any influence of PIQ, and there was found to be no effect of RCPM score on any of the measures. There were, however, significant group effects in the “free exploration” phase of the experiment: children with ASD visited fewer zones of the town and also spent less time moving around the environment. There were, however, no differences between groups in the “treasure hunt” task. The authors also performed correlational analyses between the exploration measures and the battery of clinical tasks that were administered to children. In the ASD group, stronger autistic tendencies (as measured by Achenbach’s Child Behavior Checklist; Achenbach and Edelbrock, 1983) were related to greater time spent revisiting areas that had already been explored, along with longer concomitant path lengths. The “treasure hunt” phase also revealed a negative relationship between the NEPSY-II Route Finding test and the time spent stationary in the virtual environment. There were no relationships between any of the variables in the TD group. These data therefore suggest that children with ASD spend less time actively exploring an environment, and may also re-visit those places that they have explored in a restricted and repetitive manner.

## REAL-WORLD EXPLORATION

It is clear from the virtual navigation studies detailed above that participants with ASD are often less likely to explore the experimental space as extensively as TD participants. Interestingly, this does not necessarily appear to be a phenomenon that is restricted to virtual environments, and there have been studies that also report a reduction of exploratory behavior in real-world 3D environments. Critically, this is a different behavior to that which was described in the large-scale search section of this review—exploration studies tend to be entirely unconstrained by task, and participants are simply ask to “play” in a space. It is therefore arguable whether tests of exploration are addressing navigational behaviors *per se*, although a willingness to explore the surrounding environment is clearly a prerequisite to effective navigation, and other theorists have also found this work useful in their thinking about large-scale spatial behavior in ASD.

Pierce and Courchesne (2001) studied the role of the cerebellum in exploratory behavior and recruited 14 children (aged between 2 and 8 years) with ASD. These participants were compared to a sample matched only by chronological age—since the authors were primarily concerned with neuroanatomical analyses, they did not seek to match according to ability. In their behavioral task, children were taken to a testing room that contained a number of different containers. In some of these containers were a variety of novel objects, placed so that some were visible to the child from afar and would motivate exploration and inspection. Children were simply asked by the experimenter to “go and play,” and their exploration was recorded. The authors analyzed a number of behavioral measures, including duration of exploration, number of locations explored, and the nature and quantity of motor activity. They found that children with ASD spent significantly less time exploring than controls, and also visited fewer containers. Both of these factors positively correlated with cerebellar abnormality in the ASD sample, although there was no such correlation in the TD children. Overall levels of activity did not differ notably between groups, although ASD children were more likely to engage in repetitive behaviors, and this was found to be related to cerebellar and frontal lobe volume. Data from this study therefore suggest that neural atypicalities associated with ASD may have a direct effect on the likelihood on individuals engaging with exploratory behavior.

It is possible, however, that additional factors may have limited exploration in ASD in the Pierce and Courchesne (2001) study, including how visually salient the items were. This was addressed in greater depth by Kawa and Pisula (2010), who studied the relationship between the features of objects and the exploratory behavior of children with ASD and children with Down syndrome. Nine participants with ASD (mean age: 4 years) were compared to nine children with Down syndrome and nine TD participants, matched only on chronological age. Children were introduced to a room that contained three experimental zones—each consisted of an identical layout of objects, although the visual complexity of each object (i.e., color and additional detail) was manipulated between zones. Children were simply asked to “go play” and their behavior was recorded and coded to measure exploratory activity and interaction with objects. The authors found that TD children looked at the objects more than both the ASD and Down syndrome groups (who did not differ on this measure). They also found that the two atypical groups spent less time in the zone containing the most visually stimulating objects than did the TD children. There were no group differences on any other measures, including locomotor activity or interacting with the objects themselves. These data therefore suggest that the nature of objects can affect whether or not children with ASD seek to explore them, although less time was generally spent visually exploring the environment. Critically, however, there was no difference between autistic children and those with Down syndrome on any of these measures, suggesting that these factors are not specific to the ASD profile.

## ROUTES AND MAPS

The basic exploration tasks described above were unconstrained by task. In contrast, much of our daily navigational experience concerns learning an environment in order to interact with it more efficiently in future, or to find a particular object or location. This learning may take place by means of egocentric route-based interaction with the space, or by inspecting an allocentric survey-like map. A study conducted by Caron et al. (2004) sought to examine whether people with autism would demonstrate a superiority in learning spatial layout; a hypothesis that was predicated on previous demonstrations of visuospatial peaks in laboratory tasks. They conducted a series of experiments on a cohort of 16 high-functioning participants with an ASD (mean age: 18 years), and a control group of 16 TD individuals matched on chronological age, VIQ, PIQ, and full-scale IQ (measured by either the WAIS or WISC). Each of the tasks involved the same indoor maze environment, which was constructed of plain walls that were taller than the participants, and which was diffusely lit. The tasks were conducted in the same order for each participant, and in the first they were required to learn routes through the maze. The routes differed in complexity on the basis of overall length, the number of turns, and the number of decision points (i.e., junctions at which the walker must make a navigational decision). Participants were led along the route by an experimenter and were then taken back to the start of the route and required to retrace their steps for five successive iterations (note: the paper is unclear on how participants were returned to the start of the route). Participant responses were recorded by the experimenter walking behind them, and any incorrect turns at decision points were announced by the experimenter soon afterward (participants were then told to return to the junction and were shown the correct turn). In this task there were found to be no group differences—both ASD participants and controls took more time to learn the route and made more errors as complexity increased. Furthermore, both groups appeared to learn the route at the same rate. In the second task, participants were required to reverse the route that they had learnt. This was assessed on the final response trial of the first task, whereupon participants were required to reverse the route back to the start point (i.e., there was one trial measuring this). Again there were no group differences, whilst time and errors increased with the complexity of the route.

In the third task, Caron et al. (2004) were interested in whether participants could point to unseen parts of the route, providing measures of survey knowledge and egocentric orientation within that representation. Participants were led along four routes of increasing complexity, and at the end of each they were asked to point to the start point (which was not visible from the end point). Angular accuracy of the pointing response was measured by the experimenter, and although the ASD participants tended to be more inaccurate than TD controls, there was no statistically significant difference between groups. The next tasks directly assessed the production of maps based on route experience, and participants were first required to learn a path through the maze. They were then asked to recall the route on a sheet containing a matrix of dots (free recall), and on a sheet reproducing the walls of the maze (cued recall). The free recall reproductions were qualitatively and quantitatively analyzed, revealing no significant difference between groups on accuracy or speed of reproduction (after an adjustment for speed/accuracy trade-off was made). In the cued recall task, reproductions were simply marked as pass or fail, depending on whether there was an error at a decision point. On this measure, the ASD group were shown to be more successful than the TD group, although there was no difference in drawing time.

The final task from the Caron et al. (2004) study was a test of whether participants could reproduce a route in the maze that had been learned from a map. They were given a maximum of 2 min to study a route on a small-scale map of the maze and were then provided with one opportunity to reproduce it within the maze itself. The authors found that both groups produced a comparable number of errors in the route, and executed it in a similar time. However, after an adjustment for speed-accuracy trade-off, it was found that the ASD group required less time to study the map. Overall, the data from this study therefore suggest that there are no differences between TD and ASD groups across a number of navigational tasks. However, on two components of the study that required the use of maps, participants with ASD were found to perform differently to the TD group: they were more accurate at the cued recall task of a route, and spent less time memorizing a route that was to be later reproduced. The authors argue that this was indicative of superior spatial skills in individuals with autism, although it must be noted that both of the measures that showed a group difference were scored very coarsely.

## SUMMARY

On the basis of the evidence presented in this review, there appears to be a somewhat heterogeneous picture of navigational processing in ASD. There are a number of tasks that have revealed no significant differences in navigational skills between autistic participants and controls—these skills include elements of path integration, route learning, and simple place learning. There are also a number of tasks that have revealed autistic strengths, compared to TD performance: participants with ASD have demonstrated superiority for perceptual distance matching, cued recall of routes on a map, and encoding of route information from a map. However, there are also data that show a number of navigational impairments in ASD: autistic groups were slower at learning spatial regularities, less efficient in their foraging behavior, less able to learn locations based on allocentric representations, less likely to sufficiently explore an environment, and more likely to revisit locations that they have already explored.

This overall picture does not accord with the predictions of theorists such as Reser (2011) and Baron-Cohen (2008), who have both stated that people with autism demonstrate superior abilities exploring their environment. Indeed, much of the evidence presented here is directly counter to those arguments: people with autism in fact seem to be less likely to engage in exploratory activity, and that which they do produce appears to be restricted and inefficient. On the basis of superior performance in small-scale laboratory tasks, Caron et al. (2004) also predicted that ASD would be associated with superior navigational skills, and although they mostly found an absence of group effects, their theoretical position seemed unchanged in their report. It is certainly the case that they found significant effects in favor of autistic strengths for map-drawing and map-reading tasks. However, it is interesting to note that both of these measures were not directly based on large-scale navigational behavior. Rather, one was based on a drawing measure and the other was on time spent encoding route details on a map. Furthermore, the only other autistic superiority that has been reported in this literature is for perceptual matching of distance information (Giovannini et al., 2009). It is therefore arguable that the autistic strengths that have been demonstrated are within either a small-scale visuospatial domain, or a large-scale perceptual domain. In contrast, those data that have been based on large-scale visuospatial and visuomotor behavior (perhaps the *sine qua non* of navigation) have either shown no difference between groups, or have indicated notable impairments in autistic behavior.

## REMAINING CHALLENGES

With these distinctions in mind, it seems that we must meet some remaining challenges before we can claim to have provided a comprehensive account of spatial navigational behavior in autism. The first is to appreciate the full complexity of the problem—navigational behavior is supported by a wide variety of processes that rely on perceptual, motor, and cognitive systems (for discussion, see Wolbers and Hegarty, 2010). Variability in navigational skill can therefore originate at a number of different stages, and from a number of different sources. As such, proficiency (or even superiority) in one component process may not necessarily lead to proficiency elsewhere, and performance may also be attenuated by other processes that are relatively weaker. So for example, whilst autistic strengths in attending to detail (e.g., Shah and Frith, 1993) may favor the encoding of map information, deficits in imaginal viewpoint rotation (e.g., Pearson et al., 2013) may adversely affect the ability to describe a route. With an appreciation of the size of the problem, researchers will be less likely to make the assumption that behaviors observed in one context or spatial scale will necessarily transpose to another (e.g., Montello, 1993; Smith et al., 2010).

A second related challenge is for autism researchers to investigate navigation with a greater awareness of the wider research that has been conducted on the topic. Owing to its behavioral significance, navigation has been studied in many subject fields and by using many techniques and populations—whilst it might be difficult to incorporate them all into one’s thinking, it is important to bear this breadth in mind. For instance, the neuroscience literature contains many mouse models of autistic behavior (e.g., Crawley, 2007; Moy et al., 2007), and yet human studies of navigation in ASD often take place with the participant sat at a computer monitor. It would therefore be of great benefit to conduct more translational research that attempts to create some sort of parity across the behaviors tested. Autism research could also benefit from a greater awareness of the navigational research that has been conducted in neurological patients and other developmental disorders. Neuropsychological studies of topographical disorientation have revealed a taxonomy of different navigational deficits (see Aguirre and D’Esposito, 1999), and it would be of great utility to explore the presence or absence of these component processes in ASD. Equally, research into navigational impairments in Williams Syndrome (a condition that shares a number of features with ASD) provides a range of techniques and findings that can be adopted by autism researchers (for a real world example see; Farran et al., 2010). On the other hand, studies of ASD navigation also need to take into account the research that is lacking. Unlike many other behavioral processes, developmental assays of navigation are rarely conducted with reference to existing models of navigational development, and those models that do exist (Siegel and White, 1975; Golledge et al., 1985) are rather underspecified in the light of more recent knowledge. This situation will be improved if scientists can explicitly frame their work within the context of navigation research.

Finally, aspects of autism research present more general scientific challenges that must be overcome before we can conscionably make big claims about navigation in ASD. First, as is evident from the participant details included in this review, there is great variability in the chronological ages, sample sizes, matching criteria, and intellectual level of the participants tested in these studies. This means that ASD cohorts are being compared to a variety of control groups, and themselves represent a variety of ages and ability levels. At the same time, we are applying much of this thinking to a relatively restricted portion of the autism spectrum—i.e., the high functioning individuals that are able to take part in the tasks that we devise. This means that we must be careful when extrapolating behavior to that of the broader spectrum (for an insightful discussion of this topic see; Simmons et al., 2009). Second, one could argue that autism research has been particularly dogged by a desire to create grand theories that account for all aspects of the disorder. This has been a definite product of the cognitive revolution, and the literature is replete with competing accounts of the condition that have all made an appearance in a relatively short space of time. The persuasiveness and ubiquity of these accounts may have constrained research questions, with scientists attempting to account for a variety of behaviors within artificially constrained frameworks. Navigational abilities have been inaccurately represented by grand theories of autism and we should therefore take a more careful and measured approach when attempting to characterize a very complex and multi-faceted set of behaviors within a very complex and multi-faceted population.

### Conflict of Interest Statement

The author declares that the research was conducted in the absence of any commercial or financial relationships that could be construed as a potential conflict of interest.
